# Impact of early hyperoxia on 28-day in-hospital mortality in patients with myocardial injury

**DOI:** 10.1371/journal.pone.0201286

**Published:** 2018-08-07

**Authors:** Tae Yun Kim, Dong Hoon Kim, Seong Chun Kim, Changwoo Kang, Soo Hoon Lee, Jin Hee Jeong, Sang Bong Lee, Yong Joo Park, Daesung Lim

**Affiliations:** 1 Department of Emergency Medicine, Gyeongsang National University School of Medicine and and Gyeongsang National University Hospital, Jinju, Republic of Korea; 2 Gyeongsang Institute of Health Sciences, Gyeongsang National University School of Medicine, Jinju, Republic of Korea; 3 Department of Emergency Medicine, Gyeongsang National University School of Medicine and Gyeongsang National University Changwon Hospital, Changwon, Republic of Korea; Hospital Universitari Bellvitge, SPAIN

## Abstract

**Introduction:**

Despite relevant evidence that supplemental oxygen therapy can be harmful to patients with myocardial injury, the association between hyperoxia and the clinical outcome of such patients has not been evaluated. We assessed whether early hyperoxia negatively affects outcomes in hospitalized patients with myocardial injury.

**Methods:**

This was a retrospective study conducted at a tertiary referral teaching hospital. Between January 2010 and December 2016, 2,376 consecutive emergency department patients with myocardial injury, defined as a peak troponin-I level ≥ 0.2 ng/mL, within the first 24 hours of presentation were included. The metrics used to define hyperoxia were the maximum average partial pressure of oxygen (PaO_2MAX_), average partial pressure of oxygen (PaO_2AVG_), and area under the curve during the first 24 hours (AUC_24_). The association between early hyperoxia within 24 hours after presentation and clinical outcomes was evaluated using multiple imputation and logistic regression analysis. The primary outcome was 28-day in-hospital mortality. The secondary outcomes were new-onset cardiovascular, coagulation, hepatic, renal, and respiratory dysfunctions (sequential organ failure sub-score ≥ 2).

**Results:**

Compared with normoxic patients, the adjusted odds ratios (ORs) for PaO_2MAX_, PaO_2AVG_, and AUC_24_ were 1.55 (95% confidence interval (CI) 1.05–2.27; p = 0.026), 2.13 (95% CI 1.45–3.12; p = 0.001), and 1.73 (95% CI 1.15–2.61; p = 0.008), respectively, in patients with mild hyperoxia and 6.01 (95% CI 3.98–9.07; p < 0.001), 8.92 (95% CI 3.33–23.88; p < 0.001), and 7.32 (95% CI 2.72–19.70; p = 0.001), respectively, in patients with severe hyperoxia. The incidence of coagulation and hepatic dysfunction (sequential organ failure sub-score ≥ 2) was significantly higher in the mild and severe hyperoxia group.

**Conclusions:**

Hyperoxia during the first 24 hours of presentation is associated with an increased 28-day in-hospital mortality rate and risks of coagulation and hepatic dysfunction in patients with myocardial injury.

## Introduction

Supplemental oxygen administration has been considered a fundamental therapy for patients with myocardial injury and is widely administered to patients in the setting of acute coronary syndrome. Steele described oxygen use in angina pectoris in 1900, and Levy and Barach reported a case series of four patients with acute myocardial infarction (AMI) with an improved clinical status after oxygen inhalation [1, 2]. In 2007, the American College of Cardiology/American Heart Association task force guideline recommended oxygen use for all patients with unstable angina and non-ST-elevation myocardial infarction within the first 6 hours after presentation, based on the assumption that increasing arterial oxygen tension decreases acute ischemic injury and the subsequent infarct area [3]. However, this recommendation was based on evidence from two older studies conducted in the 1970s suggesting that supplemental oxygen reduces infarct size in AMI [4, 5], and has been criticized due to a lack of evidence supporting this assumption [6].

Since the first randomized clinical trial (RCT) that showed no benefit of oxygen therapy in reducing arrhythmias or mortality was published [7], several newer studies have also reported negative effects of supplemental oxygen in patients with MI, including increased coronary vascular resistance, reduced coronary blood flow, and increased risk of mortality [8–10]. In 2014, the guidelines were changed to no longer recommend routine oxygen administration in normoxic patients with AMI [11]. The AVOID study was a multicenter RCT comparing supplemental oxygen therapy with no oxygen therapy in normoxic patients with ST elevation myocardial infarction (STEMI). The study concluded that supplemental oxygen may increase myocardial injury and infarct size at 6 months [12]. Another recent study using data from the AVOID study showed that early supplemental oxygen exposure after STEMI was associated with an increase in serum cardiac troponin-I (TnI) and creatine kinase levels [13]. Furthermore, hyperoxia has been associated with higher mortality rates in various groups including patients with stroke or traumatic brain injury, patients resuscitated from cardiac arrest, and critically ill patients in an intensive care unit [14, 15].

Despite numerous previous studies suggesting that liberal supplemental oxygen therapy may be harmful to cardiac patients and that hyperoxia is closely related to poor outcomes in critically ill patients, no study has evaluated the effect of hyperoxia in patients with myocardial injury. Therefore, we designed this study to assess the association between early hyperoxia within 24 hours of presentation and 28-day in-hospital mortality rates in non-trauma patients with myocardial injury admitted to hospital via the emergency department (ED). We also evaluated the effect of early hyperoxia on cardiovascular, coagulation, hepatic, renal, and respiratory dysfunction.

## Materials and methods

### Study design and setting

This was a single-center retrospective observational cohort study conducted between January 2010 and April 2017 at Gyeongsang National University Hospital, a tertiary referral hospital located in the south-central region of the Republic of Korea. The Gyeongsang National University hospital institutional review board approved this study with the exemption of informed consent because of the retrospective nature of the analysis. The patients who presented to our ED were all enrolled in the National Emergency Department Information System (NEDIS), which is a national database prepared by 146 emergency medical centers and managed by a government-funded national ED control agency [16, 17]. In our ED, triage nurses and attending physicians entered the patients’ data including physiologic parameters at ED arrival, symptoms, and diagnosis. Basic demographic and temporal information, treatment details including drugs and procedures, and outcomes were obtained from the hospital information system of our hospital. The input data were organized using the standard NEDIS registry format in the electronic medical record (EMR) system. The validity of all data was ensured by the use of function modules within the system before the data were saved.

### Participants

**Fig 1:**
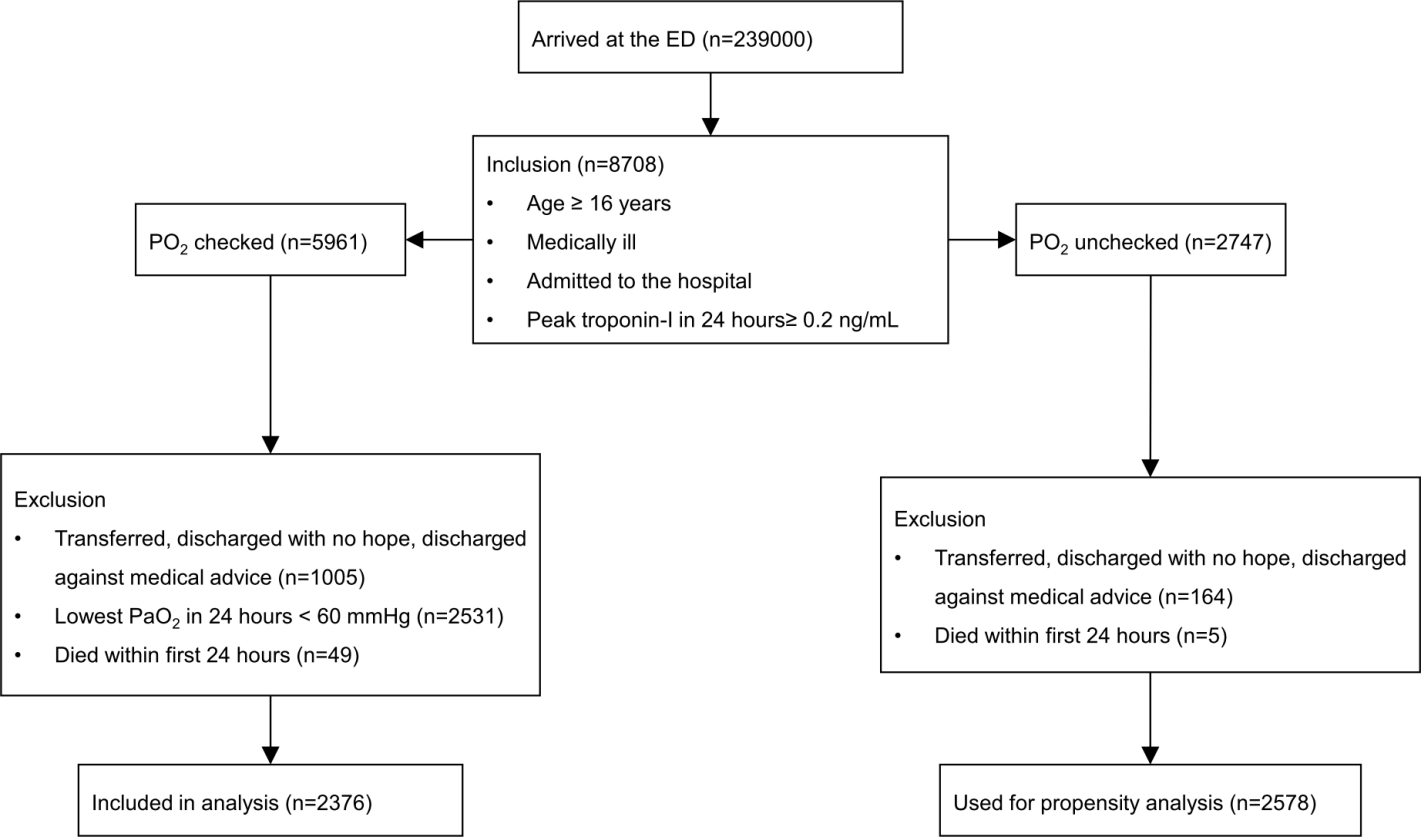
Flowchart of the inclusion and exclusion processes.

Among the consecutive ED patients ≥ 16 years of age, medically ill, and hospitalized after ED treatment, patients included in this study were those with peak TnI levels ≥ 0.2 ng/mL during the first 24 hours of presentation (assumed myocardial injury) with available partial pressure of oxygen (PaO_2_) results. We excluded patients who were transferred to other facilities after admission, who were discharged with no prospect of recovery, who left the hospital against medical advice, or who died within 24 hours of ED arrival. The purpose of this study was to compare hyperoxic patients with normoxic patients; since a single event of hypoxia within 24 hours after ED arrival might have worsened the outcomes of patients who were no longer hypoxic after ED treatment (such as supplemental oxygen), we did not include patients with a minimum PaO_2_ < 60 mmHg within 24 hours of presentation in the final analysis. Patients who were ≥ 16 years of age, medically ill, or hospitalized after ED treatment with peak TnI levels ≥ 0.2 ng/mL within the first 24 hours of presentation, but who did not have PaO_2_ results, were used for propensity analysis after exclusion according to criteria described previously. The inclusion and exclusion processes are illustrated in Fig 1.

### Data collection

Data were collected from the NEDIS registry and the EMR system of our hospital. Demographic data including age, sex, and category (diseased or injured) and physiological data including mental status described using the alert, verbal, pain, unresponsive (AVPU) scale; systolic blood pressure; heart rate; respiratory rate; body temperature; and arterial oxyhemoglobin saturation were extracted. We also extracted data from the ‘prehospital record’ and ‘list of therapeutic management’ sections of the EMR to determine whether a given patient received any supplemental oxygen therapy. The National Early Warning Score (NEWS) was calculated for each patient. Electrocardiograms obtained during the ED stay were reviewed. Temporal parameters between ED arrival and hospital discharge (date of ED arrival, admission, death, and discharge) and final outcomes of the patient (discharge, transfer, death, or other) were collected. Initial laboratory results within 2 hours of ED arrival including PaO_2_, initial complete blood count (white blood cell, hemoglobin, and platelet levels), and serum glucose, creatinine, albumin, bilirubin, and c-reactive protein (CRP) levels, were collected. The estimated glomerular filtration rate was calculated using the formula proposed by Levey et al. [18]. All PaO_2_ and TnI results within 24 hours were also collected. We collected the following additional data to evaluate new occurrences of organ dysfunction in each patient using the Sequential Organ Failure Assessment score [19]: the maximum serum bilirubin and creatinine levels and the minimum platelet count from 24 hours to 28 days; whether a given patient received vasopressors within 24 hours or after the first 24 hours of ED arrival; and whether endotracheal intubation was performed after the first 24 hours of ED arrival. We also determined whether patients underwent coronary angiography (CAG). An alternative diagnosis other than myocardial infarction (MI) was sought in each patient. The final diagnosis other than MI was extracted from the “final diagnosis” section of the EMRs. We collected clinically relevant diseases potentially related to elevated TnI levels, including pulmonary embolism, kidney disease (acute kidney injury and chronic kidney disease), aortic disease (aortic dissection, aortic aneurysm, and arterial thromboembolism), cardiac arrhythmia, active cancer, and cerebrovascular accident (ischemic and hemorrhagic stroke).

### Study outcomes

The primary outcome was the in-hospital mortality rate within 28 days of presentation. Secondary outcomes were new-onset cardiovascular, coagulation, hepatic, and renal dysfunctions (Sequential Organ Failure Assessment sub-score ≥ 2), and respiratory dysfunction after the first 24 hours of ED arrival. Since the PaO_2_/FiO_2_ ratio was not available in the majority of our patients who were not admitted to the intensive care unit, respiratory dysfunction was defined as a need for endotracheal intubation.

### Statistical analysis

Due to its retrospective nature, this study was potentially confounded by selection bias. We therefore performed a propensity analysis to investigate the extent to which execution of a blood gas test (PaO_2_ in our study; “treatment” variable) influenced the 28-day in-hospital mortality rate (dependent variable), considering the independent variables in patients who had PaO_2_ results (study group) versus those who did not (control group). An available PaO_2_ result within the first 24 hours after ED arrival was used as the treatment variable, and the 28-day in-hospital mortality rate was used as the dependent variable. In a multivariate logistic regression analysis, demographic (age and sex), physiological (NEWS), biochemical (highest levels of TnI within 24 hours, white blood cells, hemoglobin, platelets, serum glucose, creatinine, albumin, bilirubin, and CRP), and electrocardiographic (STEMI or not) data, and whether patients underwent CAG, were used as independent variables, and execution of a blood gas test (whether the patient had PaO_2_ results) was used as the dependent variable. We selected significant variables that affected the propensity of execution of a blood gas test, and the average treatment effect was calculated using propensity score matching.

We calculated the hyperoxia metrics used in a previous study [19], including the maximum PaO_2_ (PaO_2MAX_), average PaO_2_ (PaO_2AVG_), and area under the curve within the first 24 hours of admission (AUC_24_). To estimate the cumulative exposure to hyperoxia in each patient, we used the PaO_2AVG_ at the start (0 hour) and endpoint (24 hour) of the curve to calculate AUC_24_ using the trapezoid integration method. Since hyperoxia has not been defined formally, we used cutoffs based on those determined by previous studies [14,20] (normoxia, 60–120 mmHg; mild hyperoxia, 120–180 mmHg; and severe hyperoxia, > 180 mmHg) and distribution-based cutoffs in our population using the 9^th^ and 10^th^ deciles (cutoff at the 80^th^ and 90^th^ percentiles, respectively) of the PaO_2MAX_, PaO_2AVG_, and AUC_24_.

The variables with the most missing values in our dataset were the initial PaO_2_ (11.4%), initial CRP (3.1%), AUC_24_ (2.4%), and arterial oxyhemoglobin saturation (SpO_2_) (1.1%). We performed multiple imputations using chained equations (imputation number = 20) to impute missing values [21]. Predictive mean matching values from the five nearest neighbors were chosen for imputation. We assumed that the majority of the data were missing at random, because the missing laboratory results were due to the physicians’ decision to order tests depending on the patient’s condition. The AUC_24_ could be calculated only when all time-related information included in the PaO_2_ report was available. Missing time variables were caused by mechanical failure during a certain study period. As we hypothesized that the mechanism underlying these missing values was completely random, theAUC_24_ was not used for imputation and was inserted as a passive variable.

We conducted a logistic regression analysis using the imputed data, including all demographic, physiologic, laboratory, and clinical variables, and a multivariate analysis was performed using the variables that yielded a p-value < 0.001 in the univariate analysis. The adjusted odds ratios (ORs) for PaO_2MAX_, PaO_2AVG_, and AUC_24_ of the mild hyperoxia and severe hyperoxia groups compared with the normoxia group were calculated. The same analysis was performed on each secondary outcome (cardiovascular, coagulation, hepatic, renal, and respiratory dysfunctions). The Kaplan–Meier method and log rank test were used to compare the survival probabilities of the normoxia, mild hyperoxia, and severe hyperoxia groups. The χ2 test was used to determine differences in categorical data and the Mann–Whitney *U* test for differences in continuous data, as all continuous variables showed a skewed distribution. All p-values were two-sided, and a value of p < 0.05 was considered to indicate statistical significance. Analyses were conducted using Stata, version 13 (StataCorp, LP, College Station, TX, USA).

## Results

### Characteristics of the study subjects and propensity analysis

Of the 239,000 patients who presented to the ED during the study period, 5,961 met the inclusion criteria. Among them, 1,225 patients were excluded according to the exclusion criteria as follows: 1,005 were transferred, discharged with no prospect of recovery, or discharged against medical advice; 2,531 patients had a minimum PaO_2_ < 60 mmHg; and 49 did not survive 24 hours after ED arrival. Finally, 2,376 patients were included in the analysis, and 2,747 patients without PO_2_ results within 24 hours after ED arrival were eligible for propensity analysis. In total, 2,578 individuals were included in the propensity analysis after exclusion using the same criteria (Fig 1).

The baseline characteristics of the patients included in the propensity analysis are described in Table 1. Demographic and biochemical variables consistently indicated that patients in the study group were more severely ill than those in the control group (all p < 0.001). Patients in the study group had higher TnI values and more frequent incidences of STEMI and CAG (all p < 0.001). The 28-day in-hospital mortality rate was significantly higher in the study group. In the multivariate logistic regression analysis, the following significant independent variables were included in a propensity analysis: age, NEWS, level of hemoglobin, platelets, glucose, albumin, creatinine, and CRP, the highest TnI value within 24 hours, STEMI, and CAG. The average treatment effect of a PaO_2_ test on the 28-day in-hospital mortality rate was 0.7% (95% CI, −4.4 to 5.8%; p = 0.79).

**Table 1:** Baseline characteristics of control and study group used in propensity analysis.

Variable	Control group	Study group	p-value
Number of patients	2,578	2,376	
Age, years	64 (54–74)	72 (61–79)	<0.001
Male, n (%)	1,754 (68.0)	1,313 (55.3)	<0.001
National Early Waning Score	2 (0–3)	4 (2–7)	<0.001
White blood cell (x 10^3^/mm^3^)	9.36 (7.31–11.90)	9.95 (7.28–13.35)	<0.001
Hemoglobin (g/dl)	13.7 (12.4–15.0)	12.2 (10.6–13.8)	<0.001
Platelet (x 10^3^/mm^3^)	234 (197–281)	217 (163–271)	<0.001
Glucose (mg/dl)	140 (116–186)	149 (117–208)	<0.001
Albumin (g/dl)	4.1 (3.8–4.4)	3.7 (3.3–4.1)	<0.001
Bilirubin (mg/dl)	0.59 (0.41–0.85)	0.63 (0.41–1.02)	<0.001
Creatinine (mg/dl)	0.86 (0.72–1.04)	1.0 (0.76–1.50)	<0.001
eGFR (ml/min/1.73 m^2^)	85.0 (68.1–100.5)	66.7 (40.1–90.5)	<0.001
C-reactive protein (mg/dl)	1.4 (0.5–4.5)	7.6 (1.5–52.2)	<0.001
Troponin-I_MAX_ (ng/ml)	14.41 (1.23.3–16.41)	1.54 (0.43–9.43)	<0.001
ST-elevation myocardial infarction, n (%)	825 (32)	311 (13.1)	<0.001
Coronary angiography, n (%)	2,175 (84.4)	965 (40.6)	<0.001
28-day in-hospital mortality, n (%)	28 (1.1)	244 (10.3)	<0.001

eGFR, estimated glomerular filtration rate; Troponin-I_MAX_, maximum troponin-I in the first 24 hours of presentation.

Values are presented as median and interquartile range when not stated otherwise.

Continuous variables were compared by the Mann-Whitney *U* test and categorical variables were compared by χ2 test

In the study group, the median age was 72 years (IQR 61–79), and 55.3% of the patients were male (n = 1,313). In total, 311 (13.1%) patients were diagnosed with STEMI, and 965 patients (40.6%) underwent CAG. The 28-day in-hospital mortality rate was 10.3% (n = 244). The clinical characteristics of the study participants are summarized in Table 2.

**Table 2:** Baseline characteristics of study patients.

Variable		Value	Missing value, n (%)
Demographics		n = 2,376	
	Age, years	72 (61–79)	0
	Male, n (%)	1,313 (55.3)	0
Physiologic parameter			
	Systolic blood pressure (mmHg)	129 (105–150)	0
	Heart rate (per minute)	88 (75–108)	0
	Respiratory rate (per minute)	20 (20–20)	0
	Body temperature (°C)	36.6 (36.2–36.9)	2 (0.1)
	Oxyhemoglobin saturation (%)	97 (94–98)	26 (1.1)
	Consciousness (alert), n (%)	2,091 (88.0)	0
	Supplemental oxygen, n (%)	1,204 (50.7)	0
	National Early Waning Score	4 (2–7)	28 (1.2)
Biochemical parameter			
	White blood cell (x 10^3^/mm^3^)	9.95 (7.28–13.35)	5 (0.2)
	Hemoglobin (g/dl)	12.2 (10.6–13.8)	5 (0.2)
	Platelet (x 10^3^/mm^3^)	217 (163–271)	5 (0.2)
	Glucose (mg/dl)	149 (117–208)	7 (0.3)
	Albumin (g/dl)	3.7 (3.3–4.1)	7 (0.3)
	Bilirubin (mg/dl)	0.63 (0.41–1.02)	7 (0.3)
	Creatinine (mg/dl)	1.0 (0.8–1.5)	7 (0.3)
	eGFR (ml/min/1.73 m^2^)	66.7 (40.1–90.5)	7 (0.3)
	C-reactive protein (mg/dl)	0.76 (0.15–5.22)	73 (3.1)
	Troponin-I_MAX_ (ng/ml)	1.54 (0.43–9.43)	0
ST-elevation myocardial infarction, n (%)		311 (13.1)	0
Coronary angiography, n (%)		965 (40.6)	0
Alternative diagnosis, n (%)			
	Pulmonary embolism	47 (2.0)	0
	Kidney disease	147 (6.2)	0
	Aortic disease	44 (1.9)	0
	Arrhythmia	195 (8.2)	0
	Cancer	165 (6.9)	0
	Cerebrovascular accident	107 (4.5)	0
Hyperoxia metrics			
	PaO_2INI_ (mmHg)	86 (74–104)	271 (11.4)
	PaO_2MAX_ (mmHg)	97 (81–124)	0
	PaO_2AVG_ (mmHg)	90 (78–107)	0
	PaO_2MIN_ (mmHg)	79 (69–92)	0
	AUC_24_ (mmHg)	91 (78–107)	57 (2.4)
28-day in-hospital mortality, n (%)		244 (10.3)	0

eGFR, estimated glomerular filtration rate; Troponin-I_MAX_, maximum troponin-I in the first 24 hours of presentation; PaO_2INI_, initial partial pressure of oxygen; PaO_2MAX_, highest partial pressure of oxygen within first 24 hours; PaO_2AVG_, average partial pressure of oxygen within first 24 hours; AUC_24_, area under curve over the first 24 hours.

Values are presented as median and interquartile range when not stated otherwise

### Primary outcome

A univariate logistic regression analysis was conducted using all demographic, physiologic, and laboratory parameters as independent variables and the 28-day in-hospital mortality rate as a dependent variable. All variables were statistically significant except for sex, glucose level, maximum TnI level within 24 hours of ED arrival, pulmonary embolism, aortic disease, and arrhythmia. The PaO_2MAX_, PaO_2AVG_, and AUC_24_ were significant as both continuous and categorical variables (Table 3). Among the significant variables, those with a p-value < 0.001 (age, NEWS, level of white blood cells, hemoglobin, platelets, albumin, and bilirubin, estimated glomerular filtration rate, CRP level, kidney disease, cancer, and CAG) were included in the multivariate analysis with each hyperoxia variable. The adjusted ORs for PaO_2MAX_, PaO_2AVG_, and AUC_24_ (continuous variables) were 1.008 (95% CI 1.006–1.010; p < 0.001), 1.016 (95% CI 1.011–1.021; p < 0.001), and 1.014 (95% CI 1.008–1.019; p < 0.001), respectively. Using the previously defined cutoff values for mild hyperoxia and severe hyperoxia of 120 and 180 mmHg, respectively, the categorical variables PaO_2MAX_ (patients with mild hyperoxia: OR 1.55; 95% CI 1.05–2.27; patients with severe hyperoxia: p = 0.026 and OR 6.01; 95% CI 3.98–9.07, p < 0.001), PaO_2AVG_ (mild hyperoxia: OR 2.13; 95% CI 1.45–3.12; p = 0.001; severe hyperoxia: OR 8.92; 95% CI 3.33–23.88, p < 0.001), and AUC_24_ (mild hyperoxia: OR 1.73; 95% CI 1.15–2.61; p = 0.008; severe hyperoxia: OR 7.32; 95% CI 2.72–19.70, p < 0.001) all showed statistical significance. Moreover, PaO_2MAX_, PaO_2AVG_, and AUC_24_ using the 80^th^ and 90^th^ percentile cutoffs consistently showed significance (Table 4). The clinical characteristics according to hyperoxia metrics are shown in Table 5.

**Table 3:** Result of univariable analysis on imputed dataset.

Variable		Odds ratio (95% CI)	p value
Age		1.03 (1.02–1.04)	<0.001
Male		1.15 (0.89–1.51)	0.287
National Early Waning Score		1.13 (1.09–1.17)	<0.001
White blood cell		1.06 (1.03–1.08)	<0.001
Hemoglobin		0.82 (0.78–0.86)	<0.001
Platelet		1.00 (0.99–1.00)	<0.001
Glucose		1.00 (1.00–1.00)	0.136
Albumin		0.29 (0.23–0.35)	<0.001
Bilirubin		1.31 (1.21–1.42)	<0.001
Creatinine		1.12 (1.07–1.19)	<0.001
eGFR		0.99 (0.98–0.99)	<0.001
C-reactive protein		1.01 (1.00–1.01)	<0.001
Troponin-I_MAX_		0.99 (0.99–1.00)	0.214
ST elevation myocardial infarction		1.67 (1.05–2.66)	0.030
Coronary angiography		0.29 (0.21–0.41)	<0.001
Pulmonary embolism		0.81 (0.29–2.28)	0.689
Kidney disease		2.19 (1.42–3.39)	<0.001
Aortic disease		1.97 (0.91–4.30)	0.087
Arrhythmia		0.55 (0.30–1.00)	0.051
Cancer		5.53 (3.88–7.89)	<0.001
Cerebrovascular accident		1.83 (1.08–3.09)	0.024
PaO_2INI_ (mmHg)		1.01 (1.00–1.01)	0.019
PaO_2AVG_ (mmHg)		1.01 (1.01–1.02)	<0.001
PaO_2MAX_ (mmHg)		1.01 (1.01–1.01)	<0.001
AUC_24_ (mmHg)		1.01 (1.01–1.02)	<0.001
PaO_2MAX_			
	Mild hyperoxia (120–180 mmHg)	2.21 (1.58–3.08)	0.000
	Severe hyperoxia (>180 mmHg)	3.74 (1.55–9.02)	0.003
PaO_2AVG_			
	Mild hyperoxia (120–180 mmHg)	2.01 (1.45–2.80)	0.000
	Severe hyperoxia (>180 mmHg)	5.54 (3.91–7.85)	0.000
AUC_24_			
	Mild hyperoxia (120–180 mmHg)	1.84 (1.30–2.62)	0.001
	Severe hyperoxia (>180 mmHg)	4.19 (1.81–9.70)	0.001
PaO_2MAX_			
	Mild hyperoxia (136–172 mmHg)^a^	2.01 (1.45–2.80)	<0.001
	Severe hyperoxia (>172 mmHg)^b^	5.54 (3.91–7.85)	<0.001
PaO_2AVG_			
	Mild hyperoxia (111–126 mmHg)^a^	2.21 (1.58–3.08)	<0.001
	Severe hyperoxia (> 126 mmHg)^b^	3.74 (1.55–9.02)	0.003
AUC_24_			
	Mild hyperoxia (112–126 mmHg)^a^	1.84 (1.30–2.62)	<0.001
	Severe hyperoxia (>126 mmHg)^b^	4.19 (1.81–9.70)	<0.001

CI, confidence interval; eGFR, estimated glomerular filtration rate; Troponin-I_MAX_, maximum troponin-I in the first 24 hours of presentation; PaO_2INI_, initial partial pressure of oxygen; PaO_2MAX_, highest partial pressure of oxygen within first 24 hours; PaO_2AVG_, average partial pressure of oxygen within first 24 hours; AUC_24_, area under curve over the first 24 hours.

^a^ 9^th^ decile

^b^ 10^th^ decile

**Table 4:** Adjusted odds ratios of hyperoxia on 28-day in-hospital mortality.

Variable		Odds Ratio (95% CI)	p value
As continuous variable			
PaO_2MAX_		1.008 (1.006–1.010)	<0.001
PaO_2AVG_		1.016 (1.011–1.021)	<0.001
AUC_24_		1.014 (1.008–1.019)	<0.001
As categorical variable			
PaO_2MAX_			
	Mild hyperoxia (120–180 mmHg)	1.55 (1.05–2.27)	0.026
	Severe hyperoxia (>180 mmHg)	6.01 (3.98–9.07)	<0.001
PaO_2AVG_			
	Mild hyperoxia (120–180 mmHg)	2.13 (1.45–3.12)	0.001
	Severe hyperoxia (>180 mmHg)	8.92 (3.33–23.88)	<0.001
AUC_24_			
	Mild hyperoxia (120–180 mmHg)	1.73 (1.15–2.61)	0.008
	Severe hyperoxia (>180 mmHg)	7.32 (2.72–19.70)	<0.001
PaO_2MAX_			
	Mild hyperoxia (136–172 mmHg)^a^	1.68 (1.06–2.66)	0.003
	Severe hyperoxia (>172 mmHg)^b^	6.18 (4.20–9.09)	<0.001
PaO_2AVG_			
	Mild hyperoxia (111–126 mmHg)^a^	1.71 (1.08–2.72)	0.022
	Severe hyperoxia (> 126 mmHg)^b^	3.44 (2.32–5.10)	<0.001
AUC_24_			
	Mild hyperoxia (112–126 mmHg)^a^	1.82 (1.14–2.93)	0.013
	Severe hyperoxia (>126 mmHg)^b^	2.60 (1.72–3.91)	<0.001

Hyperoxia metrics were adjusted by age, National Early Warning Score, white blood cell count, hemoglobin, platelet, albumin, bilirubin, estimated glomerular filtration rate, c-reactive protein, coronary angiography, kidney disease, and cancer.

CI, confidence interval; PaO_2MAX_, highest partial pressure of oxygen within first 24 hours; PaO_2AVG_, average partial pressure of oxygen within first 24 hours; AUC_24_, area under curve over the first 24 hours.

^a^ 9^th^ decile

^b^ 10^th^ decile

**Table 5:** Clinical characteristics according to hyperoxia metrics.

Variable	PaO_2MAX_				PaO_2AVG_				AUC_24_			
	normoxia	mild hyperoxia	severe hyperoxia	p value	normoxia	mild hyperoxia	severe hyperoxia	p value	normoxia	mild hyperoxia	severe hyperoxia	p value
	<120 mmHg	120–180 mmHg	>180 mmHg		<120 mmHg	120–180 mmHg	>180 mmHg		<120 mmHg	120–180 mmHg	>180 mmHg	
N	1727	441	208		2046	297	26		2009	283	27	
Age	73 (62–79)	73 (59–80)	71 (59.5–78)	0.08	73 (62–79)	72 (59–80)	67 (46–78)	<0.05	73 (62–80)	72 (59–79)	65 (46–78.75)	<0.05
Sex, male n (%)	972 (56.3)	230 (52.2)	111 (53.4)	0.25	1136 (55.3)	167 (56.2)	10 (38.5)	0.21	1115 (55.5)	153 (54.1)	15 (55.6)	0.9
NEWS	4 (1–6)	6 (3–9)	6 (3–10)	<0.01	4 (2–7)	5 (3–9)	7 (2–11)	<0.01	4 (2–7)	6 (3–9)	5.5 (3–9)	<0.01
WBC (x 10^3^/mm^3^)	9.56 (7.22–12.61)	10.83 (7.60–14.29)	12.31 (7.90–15.88)	<0.01	9.78 (7.26–13.04)	10.96 (7.62–14.74)	11.12 (6.93–12.77)	<0.01	9.77 (7.25–13.09)	10.9 (7.61–14.27)	10.96 (8.63–15.83)	<0.01
Hemoglobin (g/dl)	12.3 (10.7–13.9)	11.7 (10.1–13.2)	12.2 (10.5–13.9)	<0.01	12.2 (10.6–13.8)	12.1 (10.5–13.7)	12.4 (10.6–13.5)	0.57	12.2 (10.6–13.8)	11.8 (10.4–13.7)	12.6 (11.3–13.5)	0.18
Platelet (x 10^3^/mm^3^)	216 (166–272)	223 (160–272)	220 (163–264)	0.94	217 (165–272)	217 (161–267)	229 (184–255)	0.94	217 (166–272)	216 (155–267)	221 (168–253)	0.83
Glucose (mg/dl)	142 (115–195)	170 (127–221)	170 (131–238)	<0.01	146 (116–203)	166 (131–225)	163 (116–207)	<0.01	146 (116–202)	175 (132–225)	171 (110–235)	<0.01
Albumin (g/dl)	3.8 (3.3–4.1)	3.6 (3.2–4.1)	3.7 (3.1–4.1)	<0.05	3.7 (3.3–4.1)	3.7 (3.2–4.1)	3.9 (3.3–4.2)	0.63	3.7 (3.3–4.1)	3.8 (3.2–4.1)	4.0 (3.3–4.2)	0.66
Bilirubin (mg/dl)	0.63 (0.41–1.02)	0.61 (0.42–1.04)	0.67 (0.43–1.02)	0.6	0.63 (0.41–1.03)	0.65 (0.43–1.01)	0.61 (0.40–0.80)	0.71	0.63 (0.41–1.03)	0.64 (0.42–0.96)	0.67 (0.40–0.99)	0.93
eGFR (ml/min/1.73 m^2^)	69.6 (43.0–92.6)	56.5 (32.3–83.5)	61.6 (39.3–86.4)	<0.01	67.3 (40.0–90.9)	61.9 (38.7–86.4)	77.4 (57.4–93.6)	0.09	67.4 (39.6–90.7)	61.2 (37.9–87.7)	77.0 (45.7–86.4)	0.18
CRP (mg/dl)	7.3 (1.5–50.8)	9.9 (1.5–53.9)	5.7 (1.6–61.1)	0.88	8.2 (1.6–54.3)	4.7 (1.1–42.0)	2.1 (0.6–7.4)	<0.01	8.5 (1.6–54.9)	4.6 (1.1–36.5)	4.1 (0.4–21.7)	<0.01
Troponin-I_MAX_ (ng/ml)	1.43 (0.41–10.45)	1.56 (0.50–8.10)	2.04 (0.59–8.91)	0.34	1.43 (0.42–9.4)	1.89 (0.58–9.7)	6.80 (1.17–23.0)	<0.05	1.39 (0.41–8.70)	1.89 (0.56–9.16)	3.28 (0.83–22.39)	<0.05
STEMI, n (%)	233 (13.5)	51 (12.9)	21 (10.1)	0.39	270 (13.2)	35 (11.8)	6 (23.1)	0.26	254 (12.6)	11 (11.7)	7 (25.9)	0.10
CAG, n (%)	759 (43.9)	157 (35.6)	49 (23.6)	<0.01	861 (41.9)	92 (31.0)	12 (46.2)	<0.01	818 (40.7)	94 (33.2)	8 (29.6)	<0.01
PE, n (%)	40 (85.1)	5 (10.6)	2 (4.3)	<0.01	44 (93.6)	3 (6.4)	0	<0.01	44 (93.6)	3 (6.4)	0	<0.05
Arrhythmia, n (%)	134 (68.7)	44 (22.6)	17 (8.7)	<0.01	165 (84.6)	27 (13.8)	3 (1.5)	<0.01	158 (83.2)	30 (15.8)	2 (1.1)	<0.01
Kidney, n (%)	95 (64.6)	37 (25.2)	15 (10.2)	<0.01	137 (83.0)	25 (15.2)	3 (1.8)	<0.01	127 (88.2)	16 (11.1)	1 (0.7)	<0.01
CVA, n (%)	60 (56.1)	26 (24.3)	21 (19.6)	<0.01	84 (78.5)	21 (19.6)	2 (1.9)	<0.01	83 (79.0)	18 (17.1)	4 (3.8)	<0.01
Aorta, n (%)	20 (45.5)	1 (2.3)	23 (52.3)	<0.01	22 (50.0)	18 (40.9)	4 (9.1)	<0.05	25 (56.8)	14 (31.8)	5 (11.4)	<0.01
Cancer, n (%)	110 (66.7)	37 (22.4)	18 (10.9)	<0.01	137 (83.0)	25 (15.2)	3 (1.8)	<0.05	132 (81.5)	27 (16.7)	3 (1.9)	<0.01
28-day in-hospital mortality, n (%)	123 (7.12)	59 (13.38)	62 (29.81)	<0.01	184 (8.96)	53 (17.85)	7 (26.92)	<0.01	183 (9.11)	45 (15.9)	8 (29.63)	<0.01
	normoxia	mild hyperoxia	severe hyperoxia	p value	normoxia	mild hyperoxia	severe hyperoxia	p value	normoxia	mild hyperoxia	severe hyperoxia	p value
	<136 mmHg	136–172 mmHg	>172 mmHg		<111mmHg	111–126 mmHg	>126 mmHg		<112 mmHg	112–126 mmHg	>126 mmHg	
N	1907	233	236		1896	242	238		1860	221	238	
Age	73 (62–79)	74 (58.75–80)	72 (59–79)	0.17	73 (63–80)	72 (58–78)	71 (58–79)	<0.01	73 (63–80)	72 (59.75–79)	71 (57–79)	<0.05
Sex, male n (%)	1068 (81.3)	120 (9.1)	125 (9.5)	0.32	1060 (80.7)	119 (9.1)	134 (10.2)	0.13	1039 (81.0)	113 (8.8)	131 (10.2)	0.41
NEWS	4 (1–7)	7 (4–10)	6 (3–10)	<0.01	4 (2–7)	5 (2–8)	6 (3–9)	<0.01	4 (2–7)	4 (2–8)	6 (3–9)	<0.01
WBC (x 10^3^/mm^3^)	9.59 (7.23–12.71)	11.71 (7.92–14.8)	12.23 (7.97–15.77)	<0.01	9.78 (7.30–13.02)	9.84 (7.14–13.45)	11.26 (7.50–14.86)	<0.01	9.85 (7.30–13.11)	9.26 (6.94–12.82)	11.24 (7.69–14.72)	<0.01
Hemoglobin (g/dl)	12.3 (10.7–13.9)	11.7 (9.8–13.0)	12.2 (10.5–13.75)	<0.01	12.3 (10.6–13.9)	11.8 (10.4–13.4)	12.2 (10.5–13.6)	<0.05	12.3 (10.6–13.9)	11.9 (10.2–13.2)	11.95 (10.5–13.7)	<0.05
Platelet (x 10^3^/mm^3^)	216 (166–272)	223 (158–272)	220.5 (163–266)	0.99	217 (164–272)	224 (174–270)	212 (155–266)	0.28	217 (164–274)	223 (171–264)	213 (155–266)	0.60
Glucose (mg/dl)	144 (115–198)	176 (131–229)	171 (131–235)	<0.01	145 (115–202)	161 (124–219)	165 (129–225)	<0.01	145 (115–202)	157 (122–214)	172 (131–226)	<0.01
Albumin (g/dl)	3.7 (3.3–4.1)	3.6 (3.2–4.1)	3.7 (3.1–4.1)	<0.05	3.7 (3.3–4.1)	3.8 (3.3–4.2)	3.7 (3.2–4.1)	0.48	3.7 (3.3–4.1)	3.7 (3.3–4.2)	3.8 (3.2–4.1)	0.71
Bilirubin (mg/dl)	0.63 (0.41–1.03)	0.62 (0.43–0.96)	0.66 (0.42–1.00)	0.92	0.64 (0.41–1.03)	0.57 (0.39–0.93)	0.68 (0.46–1.00)	<0.05	0.64 (0.42–1.03)	0.57 (0.39–0.92)	0.66 (0.44–0.97)	<0.05
eGFR (ml/min/1.73 m^2^)	69.2 (42.7–92.2)	54.5 (26.3–79.7)	61.6 (39.8–85.7)	<0.01	67.6 (41.0–90.6)	65.1 (30.4–94.5)	62.6 (40.1–86.2)	0.31	67.8 (39.8–90.7)	63.8 (35.4–90.5)	61.9 (40.1–87.3)	0.43
CRP (mg/dl)	7.5 (1.5–51.3)	9.3 (1.6–51.8)	6.7 (1.75–58.8)	0.85	8.6 (1.6–55.6)	3.9 (1.1–32.3)	4.7 (1.0–41.6)	<0.01	8.7 (1.7–56.4)	4.5 (1.1–34.2)	4.4 (0.9–41.2)	<0.01
Troponin-I_MAX_ (ng/ml)	1.47 (0.42–10.48)	1.43 (0.49–6.77)	1.98 (0.56–8.09)	0.60	1.43 (0.42–9.08)	2.06 (0.50–12.62)	1.90 (0.60–10.09)	0.08	1.33 (0.41–8.12)	2.68 (0.45–13.02)	1.92 (0.60–10.02)	<0.05
STEMI, n (%)	260 (83.6)	28 (9.0)	23 (7.4)	0.22	248 (79.7)	31 (10.0)	32 (10.3)	0.98	229 (77.9)	35 (11.9)	30 (10.2)	0.33
CAG, n (%)	1071 (75.9)	161 (11.4)	179 (12.7)	<0.01	801 (83.0)	93 (9.6)	71 (7.4)	<0.01	760 (82.6)	86 (9.3)	74 (8.0)	<0.05
PE, n (%)	41 (87.2)	4 (8.5)	2 (4.3)	0.38	42 (89.4)	2 (4.3)	3 (6.4)	0.24	42 (89.4)	3 (6.4)	2 (4.3)	0.26
Arrhythmia, n (%)	149 (76.4)	23 (11.8)	23 (11.8)	0.37	147 (75.4)	27 (13.8)	21 (10.8)	0.18	142 (74.7)	23 (12.1)	25 (13.2)	0.14
Kidney, n (%)	107 (72.8)	22 (15.0)	18 (12.2)	<0.05	108 (73.5)	25 (17.0)	14 (9.5)	<0.05	111 (77.1)	16 (11.1)	17 (11.8)	0.62
CVA, n (%)	67 (62.6)	13 (12.1)	27 (25.2)	<0.01	75 (70.1)	13 (12.1)	19 (17.8)	<0.05	74 (70.5)	12 (11.4)	19 (18.1)	<0.05
Aorta, n (%)	20 (45.5)	0 (0.0)	24 (54.5)	<0.01	20 (45.5)	4 (9.1)	20 (45.5)	<0.01	21 (47.7)	5 (11.4)	18 (40.9)	<0.01
Cancer, n (%)	128 (77.6)	18 (10.9)	19 (11.5)	0.66	121 (73.3)	20 (12.1)	24 (14.5)	0.07	118 (72.8)	22 (13.6)	22 (13.6)	0.05
28-day in-hospital mortality, n (%)	140 (7.34)	33 (14.16)	71 (30.08)	<0.01	160 (8.44)	29 (11.98)	55 (23.11)	<0.01	161 (8.66)	28 (12.67)	47 (19.75)	<0.01

PaO_2MAX_, highest partial pressure of oxygen within first 24 hours; PaO_2AVG_, average partial pressure of oxygen within first 24 hours; AUC_24_, area under curve over the first 24 hours.; NEWS, National Early Warning Score; WBC, white blood cell; eGFR, estimated glomerular filtration rate; Troponin-I_MAX_, maximum troponin-I in the first 24 hours of presentation; STEMI, ST-elevation myocardial infarction; CAG, coronary angiography; PE, pulmonary embolism; CVA, cerebrovascular accident.

Values are presented as median and interquartile range when not stated otherwise.

Continuous variables were compared by Kruskal-Wallis test and categorical variables were compared by χ2 test

### Secondary outcomes

A multivariate logistic analysis was performed to determine the effects of clinical variables found to be significant in the univariate analysis. As categorical variables, PaO_2MAX_, PaO_2AVG_, and AUC_24_ were significantly correlated with the occurrence of coagulation and hepatic dysfunction. Moreover, a higher PaO_2MAX_ was significantly associated with the occurrence of respiratory failure compared with normoxia, and other hyperoxia variables showed positive associations with respiratory failure (Table 6).

**Table 6:** Adjusted odds ratios (95% confidence interval) of hyperoxia on secondary outcomes.

Cutoff	Variable		Cardiovascular failure	Coagulation failure	Hepatic failure	Renal failure	Respiratory failure
120 and 180 mmHg	PaO_2MAX_						
		Mild hyperoxia	0.77 (0.53–1.12)	1.79 (1.12–2.85)^a^	1.18 (0.75–1.86)	0.88 (0.54–1.41)	0.85 (0.46–1.59)
		Severe hyperoxia	0.96 (0.59–1.57)	5.96 (3.77–9.42)^b^	2.90 (1.82–4.62)^b^	1.94 (1.16–3.26)^a^	3.01 (1.74–5.22)^b^
	PaO_2AVG_						
		Mild hyperoxia	1.00 (0.66–1.51)	3.00 (2.00–4.52)^b^	2.09 (1.37–3.21)^b^	1.17 (0.71–1.94)	1.31 (0.72–2.38)
		Severe hyperoxia	0.29 (0.04–2.23)	4.40 (1.5–12.91)^b^	3.58 (1.17–10.96)^a^	2.68 (0.75–9.63)	3.46 (0.98–12.28)
	AUC_24_						
		Mild hyperoxia	1.06 (0.70–1.60)	2.38 (1.54–3.68)^b^	1.67 (1.06–2.64)^a^	1.40 (0.86–2.29)	1.29 (0.70–2.39)
		Severe hyperoxia	1.07 (0.31–3.69)	5.34 (2.00–14.27)^b^	2.91 (0.95–8.90)^a^	1.56 (0.35–6.94)	1.80 (0.40–8.17)
80^th^ and 90^th^ percentile	PaO_2MAX_						
		Mild hyperoxia	0.62 (0.36–1.05)	1.60 (0.90–2.83)	0.89 (0.47–1.68)	0.64 (0.32–1.27)^a^	1.03 (0.48–2.21)
		Severe hyperoxia	0.89 (0.56–1.42)	5.41 (3.50–8.36)^b^	2.84 (1.83–4.42)^b^	2.20 (1.37–3.53)	2.85 (1.67–4.86)^b^
	PaO_2AVG_						
		Mild hyperoxia	0.65 (0.39–1.08)	1.50 (0.86–2.60)	0.90 (0.47–1.71)	1.04 (0.58–1.87)	1.38 (0.71–2.69)
		Severe hyperoxia	0.99 (0.63–1.55)	3.41 (2.20–5.28)^b^	2.72 (1.76–4.21)^b^	1.51 (0.90–2.54)	1.51 (0.81–2.82)
	AUC_24_						
		Mild hyperoxia	0.73 (0.43–1.24)	1.50 (0.83–2.71)	0.64 (0.30–1.33)	1.50 (0.86–2.61)	0.81 (0.35–1.92)
		Severe hyperoxia	1.16 (0.75–1.79)	2.99 (1.91–4.70)^b^	2.18 (1.39–3.44)^b^	1.59 (0.94–2.69)	1.48 (0.79–2.78)

All hyperoxia metrics were adjusted by variables with p < 0.05 in univariable regression.

PaO_2MAX_, highest partial pressure of oxygen within first 24 hours; PaO_2AVG_, average partial pressure of oxygen within first 24 hours; AUC_24_, area under curve over the first 24 hours.

^a^ p < 0.05

^b^ p < 0.01

### Survival analysis

**Fig 2:**
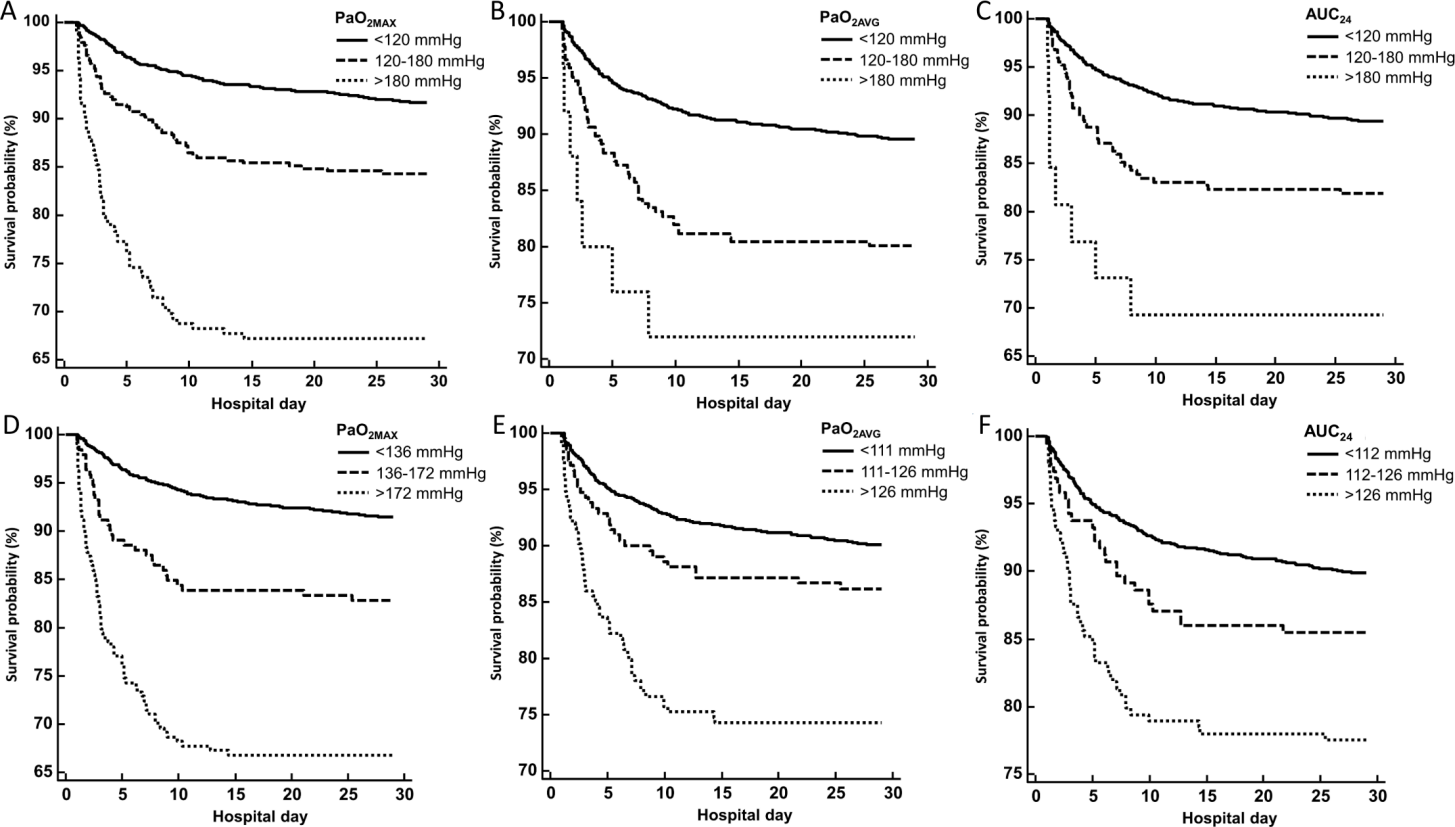
Survival analysis according to hyperoxia metrics. At the cutoffs of 120 and 180 mmHg, **A)** patients with highest partial pressure of oxygen (PaO_2MAX_) between 120 and 180 mmHg, and those with PaO_2MAX_ over 180 mmHg were associated with higher 28-day in-hospital mortality compared with normoxic patients (PaO_2MAX_ 60-120mmHg) (log rank test p<0.01). **B)** Average partial pressure of oxygen (PaO_2AVG_) and **C)** area under curve over the first 24 hours (AUC_24_) reflecting cumulative oxygen exposure were also shown to be associated with higher mortality (log rank test p<0.01 both). At the cutoffs of 80^th^ and 90^th^ percentile in each hyperoxia metrics, **D)** PaO_2MAX_, **E)** PaO_2AVG_, and **F)** AUC_24_ showed consistent associations with higher mortality, and all were statistically significant (log rank test p<0.01).

There was a tendency for mortality to increase with the severity of hyperoxia (Table 5). The hazard ratios for mild hyperoxia ranged from 1.44 to 2.15, and those for severe hyperoxia ranged from 2.46 to 4.85 (Table 7). In the Kaplan–Meier analysis, the mortality differences between the normoxia and hyperoxia groups were statistically significant regardless of the hyperoxia metrics included (all p < 0.01, log rank test) (Fig 2).

**Table 7:** Hazard ratio of hyperoxia compared with normoxia.

Variable		Hazard ratio (95% CI)
PaO_2MAX_		
	Mild hyperoxia (120–180 mmHg)	1.99 (1.43–2.77)
	Severe hyperoxia (>180 mmHg)	4.85 (3.02–7.80)
PaO_2AVG_		
	Mild hyperoxia (120–180 mmHg)	2.04 (1.39–2.99)
	Severe hyperoxia (>180 mmHg)	3.17 (0.92–10.94)
AUC_24_		
	Mild hyperoxia (120–180 mmHg)	1.81 (1.22–2.69)
	Severe hyperoxia (>180 mmHg)	3.59 (1.03–12.46)
PaO_2MAX_		
	Mild hyperoxia (136–172 mmHg)a	2.15 (1.39–3.34)
	Severe hyperoxia (>172 mmHg)b	4.79 (3.06–7.48)
PaO_2AVG_		
	Mild hyperoxia (111–126 mmHg)	1.44 (0.95–2.19)
	Severe hyperoxia (>126 mmHg)	2.95 (1.91–4.55)
AUC_24_		
	Mild hyperoxia (112–126 mmHg)	1.48 (0.96–2.29)
	Severe hyperoxia (>126 mmHg)	2.46 (1.59–3.80)

CI, confidence interval; PaO_2MAX_, highest partial pressure of oxygen within first 24 hours; PaO_2AVG_, average partial pressure of oxygen within first 24 hours; AUC_24_, area under curve over the first 24 hours.

## Discussion

In this single-center retrospective observational study, we assessed the association between early hyperoxia within the first 24 hours after presentation to the ED and clinical outcomes in medically ill hospitalized patients. The peak (PaO_2MAX_), average (PaO_2AVG_), and cumulative (AUC_24_) metrics of hyperoxia during the first 24 hours of presentation were significantly associated with poor outcomes, including the 28-day in-hospital mortality rate and occurrence of coagulation and hepatic dysfunction. Although other secondary outcome measures including cardiovascular, renal, and respiratory dysfunctions failed to reach statistical significance, they showed similar association trends (Table 7).

Two previous studies (the AVOID study and a descriptive study using data from the AVOID study) showed associations of supplemental oxygen use with increased infarct size and cardiac biomarkers. However, in terms of mortality, these studies could not verify statistical significance [12, 13]. In a recent systematic review that included a meta-analysis of the AVOID study and four other RCTs, the authors concluded there were no statistically significant differences between supplemental oxygen use and poor clinical outcomes (in-hospital mortality and infarct size) [22]. Numerous previous studies have demonstrated unfavorable outcomes of hyperoxia in critically ill patients. A meta-analysis that included 16 observational studies and one prospective before–after study demonstrated that hyperoxia may be associated with increased mortality rates in patients with stroke, traumatic brain injury, and resuscitated from cardiac arrest [14]. Additionally, a recent RCT involving intensive-care-unit patients showed that the conservative oxygen group (PaO_2_ 70–100 mmHg or SpO_2_ 94–98%) had favorable outcomes (mortality, shock, liver failure, and bacteremia) compared to those who received conventional therapy (PaO_2_ ≤ 150 mmHg or SpO_2_ 97–100%) [15]. Despite these notable findings suggesting that hyperoxia is associated with unfavorable clinical outcomes, no study has evaluated the impact of hyperoxia in cardiac patients. To the best of our knowledge, this is the first study showing a correlation between hyperoxia confirmed by blood gas analysis and clinical outcomes.

Our study population has several differences compared to those of previous studies. First, the patients’ median age (72 years) was older, and the proportion of male patients (55.3%) was lower compared to studies conducted in MI patients (range 56–63 years; proportion of males 71–80%) [23]. Next, the mean heart rate (88/minute) of our patients was much faster than that in the AVOID study population, whereas oxyhemoglobin saturation and systolic blood pressure were slightly worse (97% vs. 98% and 129 vs. 130 mmHg). Lastly, the in-hospital mortality rate in our study was much higher than that in the AVOID study (10.3% vs. 3.2%) [12].

Our study population was composed of elderly individuals and more severely ill patients compared to previous studies. Moreover, our patients were more heterogeneous and had more spontaneous MIs with intracoronary thrombi (type 1) and MIs secondary to an ischemic imbalance (type 2) compared to previous studies, which included only patients with STEMI [23]. Therefore, the present study provides a wider coverage of MI than that of previous studies, and we believe our investigation adds new strong evidence that hyperoxia or excess supplemental oxygen is harmful to cardiac patients.

Routine testing of arterial blood gas in all patients with myocardial injury (TnI ≥ 0.2 ng/mL) is not applicable in the ED setting because of invasiveness, safety, and cost. In our study population, only 68.5% (n = 5,961) of patients underwent an arterial blood gas test, and 31.5% (n = 2,747) were excluded because they did not have PaO_2_ results. While exclusion of these patients was necessary, it potentially produced selection bias. However, the results of the propensity analysis indicate that completion of a blood gas test did not affect 28-day in-hospital mortality. Patients who had PaO_2_ results (study group) showed only a 0.7% higher mortality rate compared to those who did not have PaO_2_ results (control group). We believe that this non-significant difference between the control and study groups was attributed to exclusion of hypoxia patients in our study.

This study has several limitations. First, the definition of MI in our study (TnI ≥ 0.2 ng/mL) was not sufficient to identify patients with MI according to the most recent universal definition of MI [23]. Moreover, only 40.6% (n = 965) of the patients in our study were confirmed to have AMI by CAG. The remainder comprised patients with unconfirmed type-1 MI or those with type-2 MI. The majority of the unconfirmed type-1 MI patients refused CAG. Identifying type-2 MI was difficult because invasive CAG could not be performed in patients with critical conditions, including respiratory failure, hypotension, and shock. Second, despite the results of the propensity analysis indicating minimal confounding by selection bias in this study, the risk of unidentified hidden confounders still exists. Lastly, because this was a single-center observational study conducted at a tertiary referral hospital in the Republic of Korea, our results should be validated in future multicenter prospective clinical studies.

## Conclusions

Hyperoxia during the first 24 hours of admission is associated with an increased 28-day in-hospital mortality rate and the occurrence of coagulation and hepatic dysfunction in patients with MI.
